# Supplementary material for: A transcriptomic and epigenomic cell atlas of the mouse primary motor cortex
Source: Nature. 2021 Oct 6;598(7879):103–10. doi: 10.1038/s41586-021-03500-8 (PMC8494649; doi:10.1038/s41586-021-03500-8)
Supplement: Supplementary file 1 — Evaluation of cluster replicability with MetaNeighbor. This file contains supplementary dataset, supplementary text and Appendices A and B. [file 41586_2021_3500_MOESM1_ESM.pdf]

nature portfolio

https://doi.org/10.1038/s41586-021-03500-8

Supplementary information

A transcriptomic and epigenomic cell atlas of the mouse primary motor cortex

In the format provided by the authors and unedited

Evaluation of cluster replicability with MetaNeighbor

Our paper, "An integrated multi-modal transcriptomic and epigenomic atlas of mouse primary motor cortex cell types", defines a census of transcriptomic cell types in the mouse primary motor cortex (MOp) by integrating diverse single-cell RNA-sequencing protocols. We used MetaNeighbor to measure cluster agreement across independent datasets.

In this vignette, we reproduce the MetaNeighbor analyses presented in the paper, and provide guidance for users wishing to compare their own data to the BICCN MOp annotation. The vignette, associated scripts and data can be downloaded from https://github.com/gillislab/MetaNeighbor-BICCN, MetaNeighbor is available from https://github.com/gillislab/MetaNeighbor.

Installation and required libraries

All analyses rely on the development version of MetaNeighbor. To install the latest development version, run

```
devtools::install_github("gillislab/MetaNeighbor")
```

Once MetaNeighbor is successfully installed, we load it into R's environment, along with the SingleCellExperiment package, which is used to store the transcriptomic datasets, and a script containing utility functions for visualizing results (`mn_heatmap` and `mn_heatmap_pretrained`):

```
library(MetaNeighbor)
library(SingleCellExperiment)
source("scripts/utility.R")
```

Replicability of the independent clustering of transcriptomic datasets from mouse primary cortex (MOp): applying MetaNeighbor to the full BICCN mouse MOp dataset

In this section, we reproduce the first steps of the MetaNeighbor analysis presented in the BICCN mouse MOp manuscript ("An integrated multi-modal transcriptomic and epigenomic atlas of mouse primary motor cortex cell types"). In this analysis, we quantified the agreement of the clusters obtained when clustering the seven single-cell RNA-seq datasets independently.

We start by loading a compact version of the full BICCN dataset. For memory efficiency, we reduced the dataset to highly variable genes. Alternatively, we provide the code to generate the dataset with all genes. The highly variable genes are a subset of genes that were consisently detected as variable across all datasets (using MetaNeighbor::variableGenes):

```
hvg = readLines("data/hvg.txt")

# NOT RUN: scripts used to download and build datasets
if (FALSE) {
    source("scripts/download_data.R")
    source("scripts/make_sce.R")
    dataset = readRDS("data/full_data.rds")
    # restrict to HVGs to reduce memory usage from ~25GB to ~1GB
    dataset = dataset[hvg,]
```

```
    saveRDS(dataset, "data/full_data_hvg.rds")
}

dataset = readRDS("data/full_data_hvg.rds")
dim(dataset)
```

```
## [1]    310 482712
```

To compute replicability of clusters at the subclass level, we run

```
aurocs = MetaNeighbor::MetaNeighborUS(
    var_genes = hvg, dat = dataset,
    study_id = dataset$study_id, cell_type = dataset$subclass_label,
    fast_version = TRUE, node_degree_normalization = FALSE, one_vs_best = FALSE
)
```

This function requires the user to provide a number of inputs, which are fully described in Appendix B. The basic inputs are a concatenated dataset, a set of variable genes, a set of study labels and a set of cell type labels. One important option is one__vs__best, which toggles the use of two testing frameworks for MetaNeighbor, one__vs__best or one__vs__all. One__vs__all testing computes similarity between all pairs of clusters in all datasets, and calculates an AUROC for each test cluster based on all other cells in its dataset. One__vs__best testing reports only the similarity between a training dataset and its two closest neighbors in the test dataset, calculating the AUROC as a competitive test between these two neighbors. This is a much more strict test of similarity, since the AUROC cannot be boosted by the inclusion of distant neighbors among the negatives, as is the case with one__vs__all testing.

To compare the difference between one__vs__best and one__vs__all testing, in the following step we will perform the same analysis as before, but toggle one__vs__best = TRUE.

```
aurocs_1v1 = MetaNeighbor::MetaNeighborUS(
    var_genes = hvg, dat = dataset,
    study_id = dataset$study_id, cell_type = dataset$subclass_label,
    fast_version = TRUE, node_degree_normalization = FALSE, one_vs_best = TRUE
)
```

Results are best visualized using heatmaps:

```
mn_heatmap(aurocs)
mn_heatmap(aurocs_1v1)
```

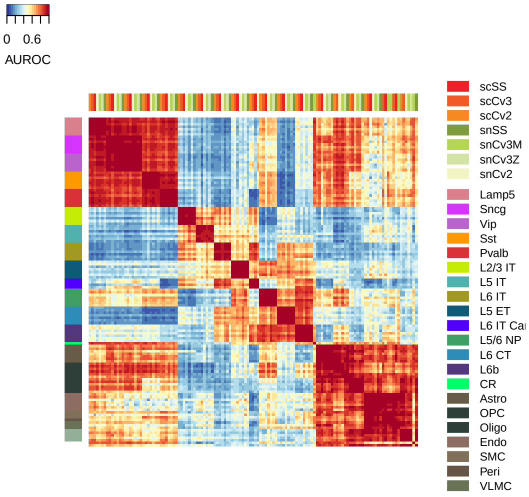

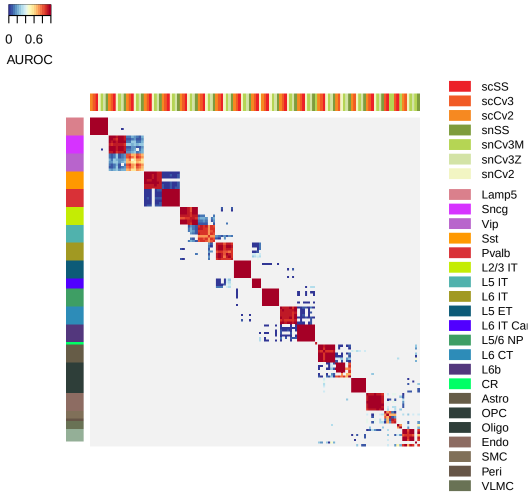

In these heatmaps, red indicates strong matching of clusters across datasests, blue indicates low matching, gray indicates that this combination of clusters was not tested (in the one-vs-best scenario, we only test the two best matching clusters against each other). The red blocks that appear along the diagonal indicate strong evidence of replicability with all clusters from a given subclass matching to each other across all datasets. Appendix A provides a more detailed explanation on how to read the heatmaps.

We can extend our analysis down to even finer resolution, comparing all pairs of clusters identified in each independent dataset:

```
aurocs_1v1 = MetaNeighbor::MetaNeighborUS(
    var_genes = hvg, dat = dataset,
    study_id = dataset$study_id, cell_type = dataset$cluster_label,
    fast_version = TRUE, node_degree_normalization = FALSE, one_vs_best = TRUE
)

mn_heatmap(aurocs_1v1)
```

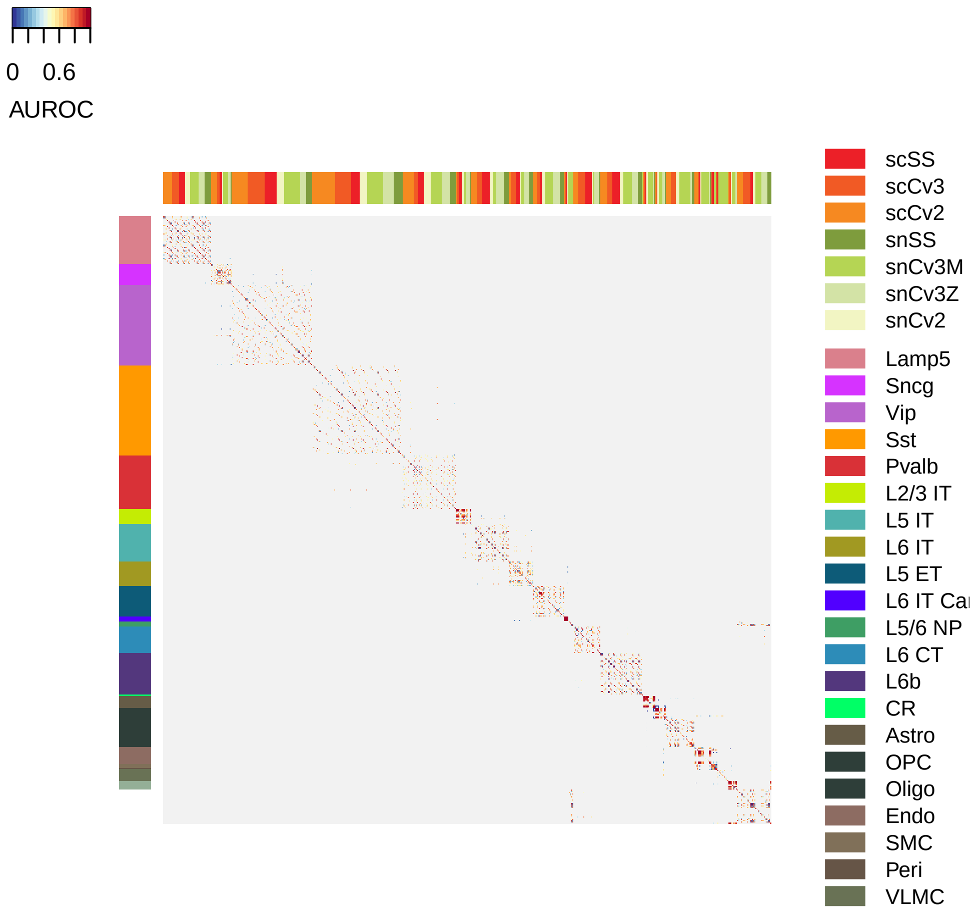

However, with this much data, replicability patterns become more complicated to visualize. In the manuscript, we quantified cluster-level replicability by counting the number of reciprocal best matches exceeding a threshold of AUROC>0.7, yielding a total of 70 cross-dataset clusters (not shown).

The analysis presented in this section is fast (less than 30s) but very memory intensive (more than 50GB of memory). How can we use this data as a reference in a more memory-efficient way?

How replicable are my clusters? Using pre-trained MetaNeighbor to compare to reference BICCN data

The BICCN MOp data is the most comprehensive and robust reference for mouse cortex currently available. Since annotation of clusters is a large bottleneck of single-cell RNA-seq analysis, users with novel brain-related data may find it helpful to compare results to the BICCN data to infer cluster labels, or to determine whether additional quality control is required. Alternatively, users with annotated clusters may wish to measure replicability of their transcriptional profiles directly.

MetaNeighbor offers the possibility of pre-training a model, which lets users run quick comparisons against a

low-memory reference dataset. These models were generated using the following code:

```r
# NOT RUN: Code used to generate pre-trained MetaNeighbor models
#          for the BICCN mouse MOp dataset
if (FALSE) {
    dataset = readRDS("data/full_data.rds")
    hvg = readLines("data/hvg.txt")

    pretrained_model = MetaNeighbor::trainModel(
        var_genes = hvg, dat = dataset,
        study_id = dataset$study_id, cell_type = dataset$subclass_label
    )
    write.table(pretrained_model,
                "pretrained_models/pretrained_biccn_mouse_mop_subclasses.txt")

    pretrained_model = MetaNeighbor::trainModel(
        var_genes = hvg, dat = dataset,
        study_id = dataset$study_id, cell_type = dataset$cluster_label
    )
    write.table(pretrained_model,
                "pretrained_models/pretrained_biccn_mouse_mop_clusters.txt")
}
```

Example 1: comparing an external dataset to the BICCN reference clusters

As a first example, we will compare the data from Paul et al. (2017) to the BICCN reference clusters. This is a dataset of GABAergic neurons from the somatosensory and motor cortices of the adult mouse brain. Cells were selected manually and RNAseq libraries were prepared using a modified Cel-seq approach, making it very technically distinct from the BICCN MOp reference. We load the data and the pre-trained model:

```r
paul_2017 = readRDS("data/paul.rds")

pretrained_biccn = as.matrix(read.table(
    "pretrained_models/pretrained_biccn_mouse_mop_clusters.txt", check.names = FALSE
))
```

We run MetaNeighbor, similar to the full dataset, except that we provide the trained model instead of highly variable genes - the HVG list is already contained in the trained model.

```r
aurocs_1v1 = MetaNeighbor::MetaNeighborUS(
    trained_model = pretrained_biccn, dat = paul_2017,
    study_id = rep("paul_2017", ncol(paul_2017)), cell_type = paul_2017$Celltype,
    one_vs_best = TRUE
)
```

Again we visualize the results with a heatmap.

```r
mn_heatmap_pretrained(aurocs_1v1)
```

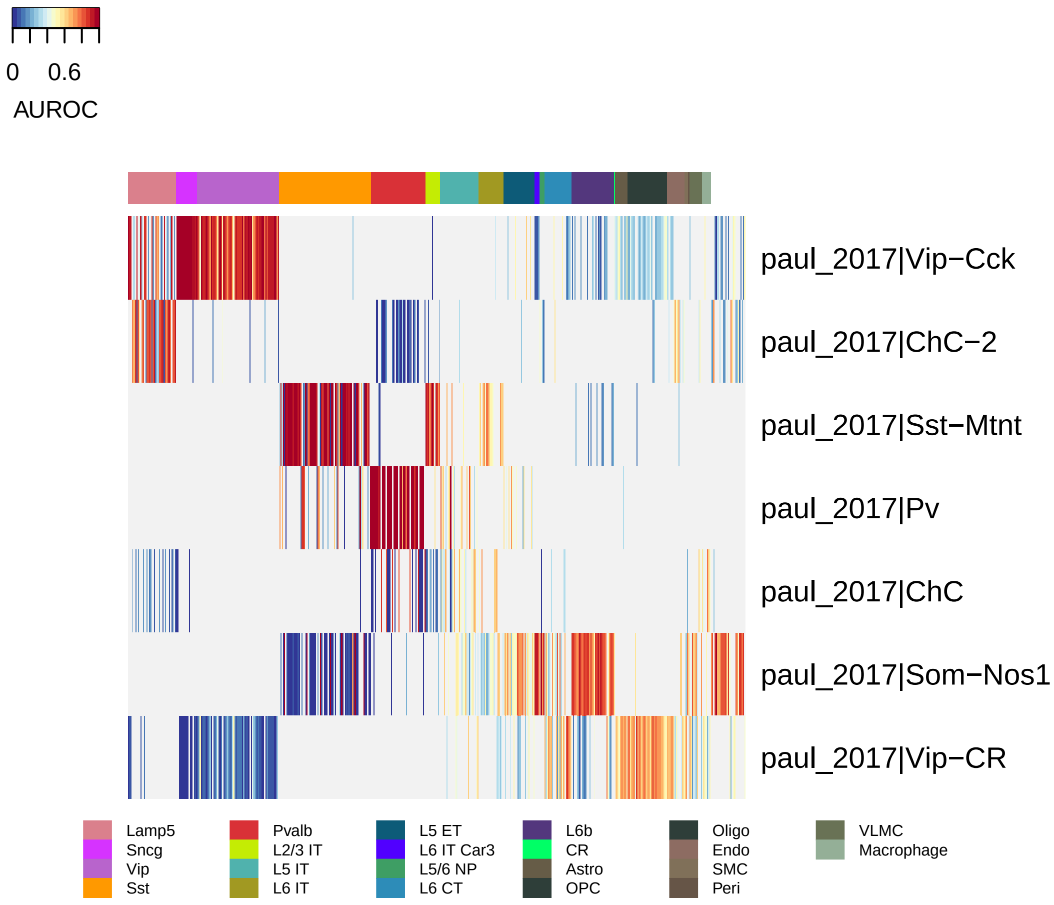

The heatmap is different: columns are train clusters, rows are test clusters, but since training and testing only occurs in one direction, the heatmap no longer has symmetrical dimensions.

To see how similar our test data is to the BICCN MOp reference in more detail, we can look at top hits for every cluster:

```
head(sort(aurocs_1v1["paul_2017|ChC",], decreasing = TRUE), 10)
```

```
##    snSS|Pvalb Vipr2  snCv3Z|Pvalb Vipr2   scCv2|Pvalb Vipr2    scSS|Pvalb Vipr2
##           0.9177885           0.9064904           0.8911058           0.8843750
##   snCv2|Pvalb Vipr2  snCv3M|Pvalb Vipr2   scCv3|Pvalb Vipr2 snCv3M|Macrophage_2
##           0.8247596           0.8221154           0.8096154           0.7688492
## scCv3|L6 IT Sulf1_1   scSS|L5 IT Tcap_2
##           0.7256434           0.6997596
```

```
head(sort(aurocs_1v1["paul_2017|Som-Nos1",], decreasing = TRUE), 10)
```

```
##    scSS|Sst Chodl  snCv3M|Sst Chodl   scCv3|Sst Chodl   snCv2|Sst Chodl
##         0.9977467         0.9969165         0.9963235         0.9953748
```

```
##   scCv2|Sst Chodl  snCv3Z|Sst Chodl    snSS|Sst Chodl  snCv3M|L5/6 NP_1
##         0.9951376         0.9951376         0.9949004         0.9773897
## scCv2|L6 NP Trh_2 snCv3M|L6b Rprm_2
##         0.9557675         0.9377298
```

We recover expected top hits for these two cell types (ChC = Chandelier, Som-Nos1 = Long projecting interneurons). There are also "unexpected" hits due to imbalance in dataset composition which are normally filtered out by swapping train and test (i.e., reciprocal testing), but they have lower AUROC values than the true hits.

Example 2: comparing one of the BICCN datasets to the BICCN reference subclasses

To further illustrate differences in memory consumption and the importance of swapping test and train, we compare one of the BICCN datasets with the pre-trained model. We chose to use the 10X cells v2 dataset, because it's the largest dataset. We subset our compact dataset to 10Xv2 cells (the full version can be built from the dowload_data.R and make_sce.R scripts), and load the pre-trained model:

```
tenx_cells = dataset[, dataset$study_id == "scCv2"]

pretrained_biccn = as.matrix(read.table(
    "pretrained_models/pretrained_biccn_mouse_mop_subclasses.txt", check.names = FALSE
))
```

We run MetaNeighbor against the pre-trained BICCN subclasses:

```
aurocs_1v1 = MetaNeighbor::MetaNeighborUS(
    trained_model = pretrained_biccn, dat = tenx_cells,
    study_id = rep("scCv2", ncol(tenx_cells)),
    cell_type = tenx_cells$subclass_label, one_vs_best = TRUE
)

mn_heatmap_pretrained(aurocs_1v1)
```

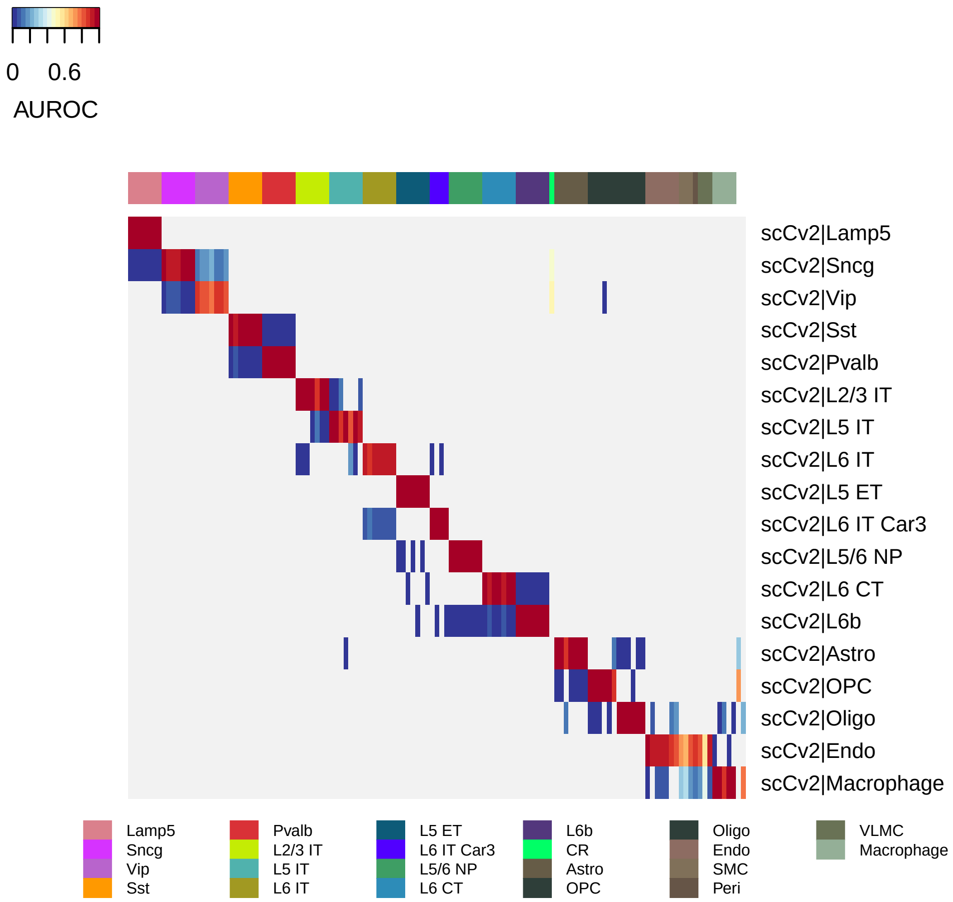

We find almost perfect replicability structure, except for non-neurons, for example endothelial cells (Endo), which map to training non-neuronal clusters not present in the 10X v2 dataset, such as vascular and leptomeningeal cells (VLMC).

Again we can investigate top hits:

```r
head(sort(aurocs_1v1["scCv2|L6 IT Car3",], decreasing = TRUE))
```

```
## scCv2|L6 IT Car3 snCv2|L6 IT Car3 scCv3|L6 IT Car3  scSS|L6 IT Car3
##       0.99998776       0.99996158       0.99992846       0.99864419
##      scCv3|L6 IT     snCv3M|L6 IT
##       0.10342055       0.08925864
```

If cell type composition is balanced across datasets, as in the example above, we find that replicability scores are well captured by one-directional testing and training. Also, if the population is present in the test dataset but is missing in some of the reference datasets we correctly find that there are no strong hits. However, when there are populations in the training data that aren't in the testing data, these can show up as incorrect strong matches:

```r
head(sort(aurocs_1v1["scCv2|Endo",], decreasing = TRUE), 10)
```

```
##   scCv2|Endo snCv3M|Endo  scCv3|Endo  snCv2|Endo snCv3M|VLMC   scSS|Endo
##    0.9635843   0.9444217   0.9243138   0.9228041   0.9134615   0.9058920
## snCv3Z|Endo snCv3M|Peri  scCv3|VLMC   snSS|Endo
##    0.8740187   0.8636116   0.8327878   0.8320972
```

Directly comparing the output from the pretrained model to MetaNeighbor's usual output can help identify incorrect strong matches. We run MetaNeighbor using the full dataset below:

```r
ref_aurocs = MetaNeighbor::MetaNeighborUS(
    var_genes = hvg, dat = dataset,
    study_id = dataset$study_id, cell_type = dataset$subclass_label,
    fast_version = TRUE, one_vs_best = TRUE
)

ref_aurocs = ref_aurocs[rownames(aurocs_1v1), colnames(aurocs_1v1)]
```

We compare the two score matrices and look for outliers, in particular cluster comparisons that have high scores in the pre-trained model but low scores with the full model:

```r
comparison_names = expand.grid(MetaNeighbor::getCellType(rownames(ref_aurocs)),
                               colnames(ref_aurocs),
                               stringsAsFactors = FALSE)
comparison_names = paste(comparison_names[,1], comparison_names[,2], sep = " vs ")
is_outlier = aurocs_1v1 > 0.8 & ref_aurocs < 0.5

par(mfrow = c(1,2), mar=c(5,4,2,2)+0.1)
plot(ref_aurocs, aurocs_1v1, pch = 16,
     xlab = "AUROC full model", ylab = "AUROC pretrained")
rect(0.3, 0.8, 0.6, 0.9, lty = "dashed")

plot(ref_aurocs, aurocs_1v1, xlim = c(0.3, 0.6), ylim = c(0.8, 0.9), pch=16, cex = 1,
     xlab = "AUROC full model", ylab = "AUROC pretrained")
text(ref_aurocs[is_outlier], aurocs_1v1[is_outlier]-0.01, comparison_names[is_outlier],
     cex = 1)
```

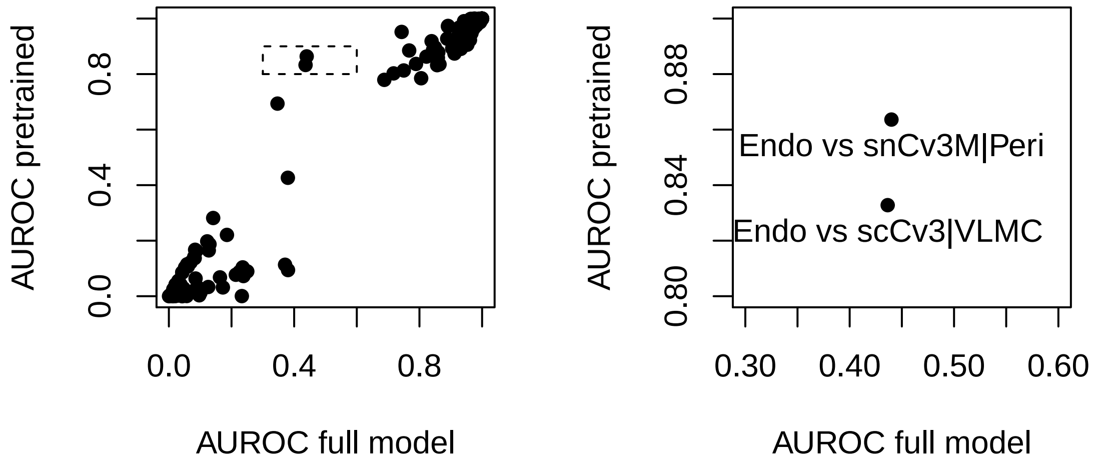

This shows that we overestimate AUROCs when populations are missing in the test dataset compared to the reference (Peri and VLMC). There are two solutions to this problem: ignore "impossible hits" by filtering out known missing populations from the training data, or by reverting to the reciprocal test-train framework rather than using the pre-trained models, though we note that this requires more memory.

Performance summary

Below we list the amount of memory needed and the run times for each of the analyses presented in this vignette.

	Full BICCN	Full BICCN (HVG only)	Pretrained BICCN - Paul 2017	Pretrained BICCN - BICCN 10X V2 cells
Dataset memory usage	25 GB	< 1 GB	0.1 GB	7 GB
MetaNeighbor memory usage	+10-15 GB	+10-15 GB	+ 0.1 GB	+ 4 GB
MetaNeighbor run time	30-40s	20-25s	< 1s	10s

Run times are low overall and should not be a concern. Reducing the BICCN data to HVGs makes MetaNeighbor analyses tractable to machines with a medium amount of memory (MetaNeighbor memory usage is overestimated due to R's tendency to not use garbage collection when memory is available). When using the pre-trained model, the amount of memory depends on the size of the testing data.

Appendices

Appendix A: Interpretation of one_vs_best heatmaps

The heatmap above shows the results of MetaNeighbor tests for human, marmoset and mouse GABAergic subclasses from the BICCN Non-Human Primate working group. Dark red 3x3 blocks along the diagonal indicate high similarity among the three datasets. We discuss how to interpret this figure in more detail in the following.

MetaNeighbor measures the similarity between groups of cells based on gene expression patterns. It is formalized as a supervised learning task by treating one group of cells as a training set, and reporting test performance for other cell groups as the area under the receiver operating characteristic curve (AUROC). In the heatmap, each column shows the performance for a single training group across three test datasets. Colors indicate the train-test performance, where red is high, yellow is random, and blue is low. AUROCs are computed between the two closest neighbors in the test dataset, where the closer neighbor will have the higher score. All others are test groups shown in gray (NA). Thus, each column contains 6 non-NA values.

The first column contains test results for human Lamp5, indicated by the colored bars along the outside of the heatmap. Here we've labeled the results for all tests with a number and a letter: numbers indicate the test dataset, where 1 is human, 2 is marmoset and 3 is mouse; letters indicate closest (a) and second-closest (b) neighboring groups.

The first test is circular: if we look within the human dataset (1) we find that Lamp5 is its own closest neighbor (1a), with Sncg as the next-closest neighbor (1b) and that these two groups are very distinct from one another (AUROC>0.95). The results are identical in marmoset, where the closest neighbor is Lamp5 (2a), and the second closest is Sncg (2b). Mouse results (3) are slightly different: while mouse Lamp5 is the closest neighbor (3a) the second-closest is mouse Vip (3b). Overall, the high AUROCs for these corresponding types suggests that there is a conserved Lamp5 gene expression signature.

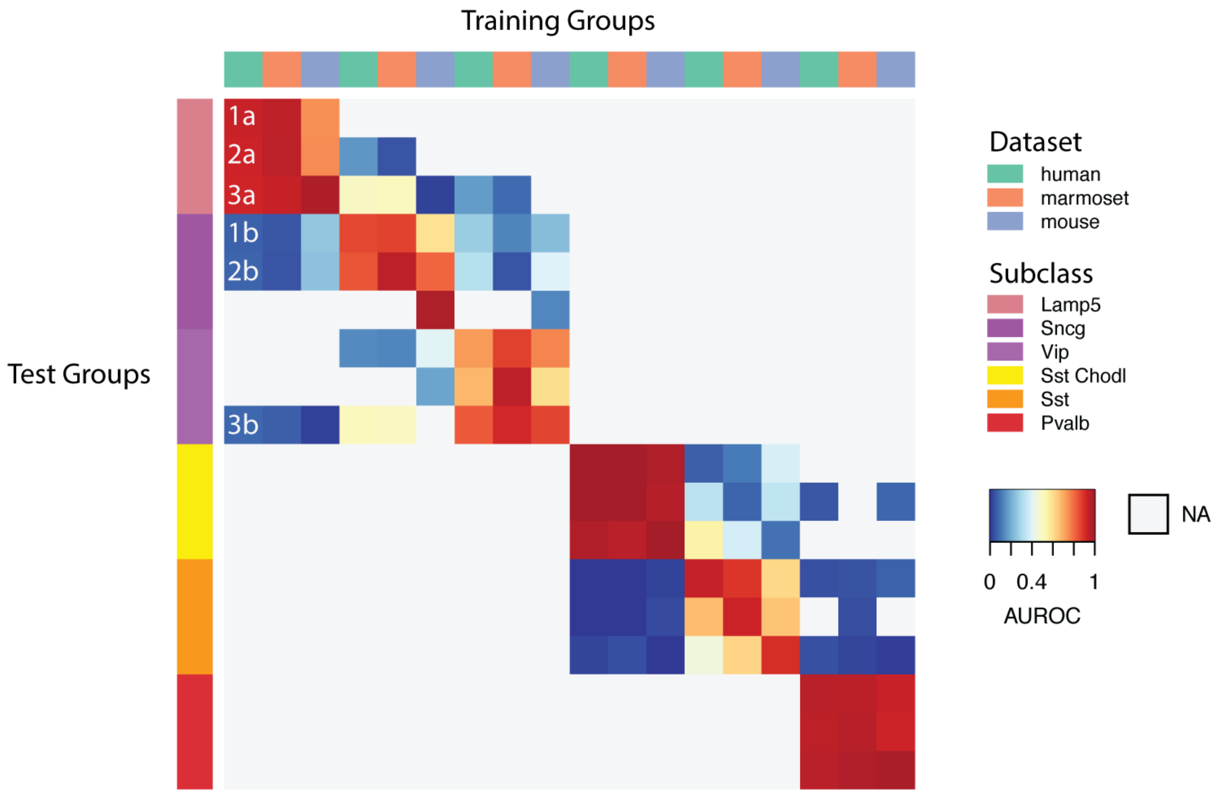

Figure 1: Heatmap interpretation

Appendix B: MetaNeighbor parameters

Below we provide a detailed description of MetaNeighbor's parameters:

- var_genes: A vector of genes.
- dat: A SingleCellExperiment object where expression data for all datasets can be found as a merged matrix in the first assay slot.
- study_id: A vector of labels indicating the study of origin for each cell.
- cell_type: A vector of labels indicating the transcriptomic identity of each cell.

There are also three optional parameters to specify:

- fast_version: This parameter toggles the use of the legacy MetaNeighborUS function, which is not advised for datasets with >10K cells. The fast version of MetaNeighbor approximates the legacy function, but fully parallelizes and vectorizes neighbor voting using matrix multiplication of ranked cell profile vectors.
- node_degree_normalization: This parameter toggles the use of node degree normalization. This parameter was initially designed to mitigate the impact of differential transcriptome coverage across cell clusters, such that those with a greater number of non-zero genes would not be universally 'loved' by all others. However, we have since found that good variable gene selection and paired testing/training is sufficient to avoid this issue. We advise setting this to FALSE for most applications, as it can create artifacts associated with cell composition differences between datasets.
- one_vs_best: This parameter toggles the use of two testing frameworks for MetaNeighbor, one_vs_best or one_vs_all, and is currently only compatible with the fast version of MetaNeighborUS. One_vs_all testing is equivalent to the legacy function of MetaNeighborUS, which computes similarity between all pairs of clusters in all datasets, and calculates an AUROC for each test cluster based on all other cells in its dataset. One_vs_best testing reports only the similarity between a training dataset and its two closest neighbors in the test dataset, calculating the AUROC as a competitive test between these two neighbors. This is a much more strict test of similarity, since the AUROC cannot be boosted by the inclusion of distant neighbors among the negatives, as is the case with one_vs_all testing.
