# Supplementary material for: A transcriptomic and epigenomic cell atlas of the mouse primary motor cortex
Source: Nature. 2021 Oct 6;598(7879):103–10. doi: 10.1038/s41586-021-03500-8 (PMC8494649; doi:10.1038/s41586-021-03500-8)
Supplement: Supplementary file 2 — Reporting Summary [file 41586_2021_3500_MOESM2_ESM.pdf]

nature research

Corresponding author(s): Eran Mukamel, Hongkui Zeng

Last updated by author(s): Mar 19, 2020

Reporting Summary

Nature Research wishes to improve the reproducibility of the work that we publish. This form provides structure for consistency and transparency in reporting. For further information on Nature Research policies, see Authors & Referees and the Editorial Policy Checklist.

Statistics

For all statistical analyses, confirm that the following items are present in the figure legend, table legend, main text, or Methods section.

n/a	Confirmed	
☐	☒	The exact sample size (*n*) for each experimental group/condition, given as a discrete number and unit of measurement
☐	☒	A statement on whether measurements were taken from distinct samples or whether the same sample was measured repeatedly
☐	☒	The statistical test(s) used AND whether they are one- or two-sided<br>*Only common tests should be described solely by name; describe more complex techniques in the Methods section.*
☐	☒	A description of all covariates tested
☐	☒	A description of any assumptions or corrections, such as tests of normality and adjustment for multiple comparisons
☐	☒	A full description of the statistical parameters including central tendency (e.g. means) or other basic estimates (e.g. regression coefficient) AND variation (e.g. standard deviation) or associated estimates of uncertainty (e.g. confidence intervals)
☐	☒	For null hypothesis testing, the test statistic (e.g. *F*, *t*, *r*) with confidence intervals, effect sizes, degrees of freedom and *P* value noted<br>*Give P values as exact values whenever suitable.*
☒	☐	For Bayesian analysis, information on the choice of priors and Markov chain Monte Carlo settings
☒	☐	For hierarchical and complex designs, identification of the appropriate level for tests and full reporting of outcomes
☐	☒	Estimates of effect sizes (e.g. Cohen's *d*, Pearson's *r*), indicating how they were calculated

Our web collection on statistics for biologists contains articles on many of the points above.

Software and code

Policy information about availability of computer code

Data collection

Software for mapping and analysis of transcriptomic and epigenomic datasets is fully described in the Methods.

SMART-Seq v4 (SSv4) Ultra Low Input RNA Kit for Sequencing (Takara Cat# 634894)

Data analysis

Software for mapping and analysis of transcriptomic and epigenomic datasets is fully described in the Methods

STAR v2.5.3
https://github.com/epiviz/miniatlas,
https://github.com/AllenInstitute/scrattch.hicat,
https://github.com/r3fang/SnapTools,
https://github.com/lhqing/cemba_data (documentation: cemba-data.rtfd.io)
https://github.com/gillislab/MetaNeighbor-BICCN,
https://github.com/welch-lab/liger,
https://github.com/mukamel-lab/SingleCellFusion
http://epiviz.nemoanalytics.org/biccn_mop,
CONOS(Barkas et al. 2018): https://github.com/kharchenkolab/conos
Bismark (Krueger and Andrews, 2011)

For manuscripts utilizing custom algorithms or software that are central to the research but not yet described in published literature, software must be made available to editors/reviewers. We strongly encourage code deposition in a community repository (e.g. GitHub). See the Nature Research guidelines for submitting code & software for further information.

Data

Policy information about availability of data

All manuscripts must include a data availability statement. This statement should provide the following information, where applicable:

- Accession codes, unique identifiers, or web links for publicly available datasets
- A list of figures that have associated raw data
- A description of any restrictions on data availability

The BICCN MOp data (RRID:SCR_015820) can be accessed via the NeMO archive (RRID:SCR_002001) at accession: https://assets.nemoarchive.org/dat-ch1nqb7. Visualization and analysis resources: NeMO analytics: https://nemoanalytics.org/, Genome browser: https://brainome.ucsd.edu/annoj/BICCN_MOp/, Epiviz browser: https://epiviz.nemoanalytics.org/biccn_mop.

Field-specific reporting

Please select the one below that is the best fit for your research. If you are not sure, read the appropriate sections before making your selection.

☒ Life sciences ☐ Behavioural & social sciences ☐ Ecological, evolutionary & environmental sciences

For a reference copy of the document with all sections, see nature.com/documents/nr-reporting-summary-flat.pdf

Life sciences study design

All studies must disclose on these points even when the disclosure is negative.

Sample size

Our study focuses on differences between major brain cell populations, which are highly conserved across individual mice. Sample size (number of animals) was determined by the experimental requirements for collection of sufficient tissue for each assay.

For RNA-seq data, we collected tissue from a total of 45 animals for SMART-seq cells, 10 for SMART-seq nuclei, and 3~12 for various 10x platforms. We used relatively larger number of animals for SMART-seq due to use of specific cre-lines and layer specific dissections. Due to highly sensitive gene detection provided by the SMART-seq platform, we collected about ~6000 cells for both cells/nuclei, targeting at least 5 cells for any cell types with abundance more than 0.1%. Our previous studies suggest that highly distinct cell types can be detected with just 5 cells, while more subtle cell type differences can be detected with 20~50 cells. For 10x, we collected at least 100,000 cells for each platform, targeting at least 100 cells for any cell types with abundance more than 0.1%.

For collection of single nuclei by FACS for the epigenomic datasets (snmC-seq and snATAC-seq), we collected tissue from 6-23 individual mice for each sub-region of MOp (different anterior-posterior levels). Larger numbers of mice were used for relatively small subregions, which ensured that at least 2,500 single neuronal nuclei (NeuN+) were obtained from each sample. We collected tissue from two independent pools of animals (biological replicates) to calibrate inter-sample variability due to inter-individual differences.

In no case did we observe differences between individual animals or batches that were similar in magnitude to the reported cell type differences. The number of cells collected was determined by specific limitations of each data modality, and the effect of this sample size was extensively analyzed as part of the paper (Figure 6).

Data exclusions

Low quality cells and putative doublets were excluded based on criteria that are described in detail in the Methods.

SMART-seq: Cells that met any one of the following criteria were removed: < 100,000 total reads, < 1,000 detected genes (CPM > 0), < 75% of reads aligned to the genome, or CG dinucleotide odds ratio > 0.5.

10x RNA-seq: For scRNA datasets, we excluded neurons with fewer than 2000 detected genes and non-neuronal cells with fewer than 1000 detected genes; for snRNA datasets, we excluded neurons with fewer than 1000 detected genes and non-neuronal cells with fewer than 500 detected genes. Doublets were identified using a modified version of the DoubletFinder algorithm and removed when doublet score > 0.3. After clustering of individual datasets, we also removed some clusters that we believed were driven by technical artifacts: clusters with strong markers indicating their regional identities outside of MOp, rare doublet clusters that were not captured by the doublet score but contain the markers from multiple highly distinct cell types (e.g. neurons and non-neuronal types), and low quality clusters showing significant gene loss, but no up-regulated genes relative to another similar cluster. Cells from these clusters were eliminated from downstream integrative analysis. A full description of the procedure for exclusion of artifactual clusters is provided in Methods in the section "Clustering individual datasets".

snATAC-seq: We excluded any single cells that had fewer than 1,000 unique fragments or a TSS enrichment of <10 for any sample sets. We used Scrublet (RRID:SCR_018098)52 to remove potential doublets for every sample set

snmC-seq: We filtered the cells based on quality metrics: 1) The rate of bisulfite non-conversion as estimated by the rate of methylation at CCC positions (mCCC) < 0.03. mCCC rate reliably estimates the upper bound of bisulfite non-conversion rate8, 2) overall mCG rate > 0.5, 3) overall mCH rate < 0.2, 4) total final reads (combining R1 and R2) > 500,000, 5) Total mapping rate (using Bismark54) > 0.5.

In addition, during the RNA consensus clustering analysis, we excluded clusters as follows (this text is in the Methods):

Removal of low-quality and doublet-driven clusters. We performed differential gene expression analysis between every pair of clusters within each subclass. If any cluster had ≤2 up-regulated genes (fold-change>2, FDR<0.01, with additional penetrance and odds ratio criteria described in Method transcriptome analysis section) compared to another cluster, and had a substantially lower average number of detected genes per cell, we flagged the cluster as low-quality and removed it from further analysis. Next, if the up-regulated genes between any two

	clusters within a subclass were predominantly marker genes for a different subclass, and one of the clusters had significantly higher average genes detected per cell and UMI count, we flagged the cluster as a potential doublet cluster and removed it from further analysis. These criteria led to the exclusion of 8.3% of all cells, the vast majority of which came from the two 10x v3 datasets (scRNA 10X v3 A, snRNA 10X v3 B). While the 10X v3 platform boosts the gene detection for good cells, it does the same to damaged cells or debris, leading to an elevated number of clusters that were excluded for these datasets.
Replication	Findings in each modality were extensively compared across biological replicates (at least 2 replicates of each experiment). We did not observe any disagreement between replicates in terms of the biological conclusions, such as the identity of cell types. In addition, we extensively characterize the multimodal correspondence and concordance of the datasets, providing robust validation of cell types.<br><br>For the snmC and snATAC data, replicates from the same brain region are co-clustered compared to samples from other brain regions. Within each cluster, we calculated Pearson correlations between each replicates, and found that all replicates are highly conserved (Pearson corr. > 0.95).
Randomization	Not applicable. Our study does not compare treatment and control groups. Instead we focus on characterizing the cell types that are present in untreated adult mice. Single cell sequencing provides a random sample of cells derived from the source tissue.
Blinding	Not applicable. There were no treatment and control groups, and no pre-defined hypotheses regarding cell type identity.

Reporting for specific materials, systems and methods

We require information from authors about some types of materials, experimental systems and methods used in many studies. Here, indicate whether each material, system or method listed is relevant to your study. If you are not sure if a list item applies to your research, read the appropriate section before selecting a response.

Materials & experimental systems

n/a	Involved in the study
☒	☐ Antibodies
☒	☐ Eukaryotic cell lines
☒	☐ Palaeontology
☐	☒ Animals and other organisms
☒	☐ Human research participants
☒	☐ Clinical data

Methods

n/a	Involved in the study
☒	☐ ChIP-seq
☒	☐ Flow cytometry
☒	☐ MRI-based neuroimaging

Animals and other organisms

Policy information about studies involving animals; ARRIVE guidelines recommended for reporting animal research

Laboratory animals	Salk: Mouse (mus musculus), strain C57BL6/J (RRID:IMSR_JAX:000664), both males and females, 53-63 days of postnatal age. Male C57BL/6J mice were purchased from Jackson laboratories at 8 weeks of age and maintained in the Salk animal barrier facility on 12-hr dark-light cycles with controlled temperature (20-22 Celcius range) and humidity (30-70% range), and food ad libitum for one week before dissection.<br><br>Allen Institute: All procedures were carried out in accordance with Institutional Animal Care and Use Committee protocols at the Allen Institute for Brain Science. Mice were provided food and water ad libitum and were maintained on a regular 12-h day/night cycle at no more than five adult animals per cage. Ambient temperature was set to 72°F and relative humidity was set to 40%.<br><br>Broad Institute: Animals were group housed with a 12-hour light-dark schedule and allowed to acclimate to their housing environment for two weeks post arrival. Ambient temperature was set to 70°F ± 2°F and relative humidity was set to 40% ± 10%. All rooms are on 12/12 hour light/dark cycle.
Wild animals	Not applicable. No wild animals were used in this study.
Field-collected samples	Not applicable. No field collected samples were used in this study
Ethics oversight	All procedures at the Allen Institute were carried out in accordance with Institutional Animal Care and Use Committee protocols at the Allen Institute for Brain Science.<br><br>All procedures involving animals at MIT were conducted in accordance with the US National Institutes of Health Guide for the Care and Use of Laboratory Animals under protocol number 1115-111-18 and approved by the Massachusetts Institute of Technology Committee on Animal Care. All procedures involving animals at the Broad Institute were conducted in accordance with the US National Institutes of Health Guide for the Care and Use of Laboratory Animals under protocol number 0120-09-16.<br><br>Experiments conducted at The Salk Institute in accordance with the US National Institutes of Health Guide for the Care and Use of Laboratory Animals under protocol number 18-00006 and approved by the Institutional Animal Care and Use Committee.

Note that full information on the approval of the study protocol must also be provided in the manuscript.
