# Supplementary material for: A transcriptomic and epigenomic cell atlas of the mouse primary motor cortex
Source: Nature. 2021 Oct 6;598(7879):103–10. doi: 10.1038/s41586-021-03500-8 (PMC8494649; doi:10.1038/s41586-021-03500-8)
Supplement: Supplementary file 4 — This zip folder contains the following 10 files: Supplementary Table 1: Sample parameters List of datasets, number of cells, and other parameters of each dataset. Data from this study are available via the Neuroscience Multi-omics Archive (NEMO, RRID:SCR_016152) at https://assets.nemoarchive.org/dat-ch1nqb7. Supplementary Table 2: Multimodal integration cluster assignments List of all cells with cluster assignments based on 3 computational methods for integrated analysis (RNA consensus, SingleCellFusion, LIGER). Supplementary Table 3: Cell type proportions per dataset Proportion of each cell type within each major cell class for each transcriptomic modality. Supplementary Table 4: Cluster analysis for individual datasets. Cluster analysis and metadata for each dataset on its own. Eight individual files:1. S4a - scRNA SMART 2. S4b - scRNA 10x v3 A 3. S4c - scRNA 10x v2 A 4. S4d - snRNA SMART 5. S4e - snRNA 10x v3 B 6. S4f - snRNA 10x v3 A 7. S4g - Open chromatin (ATAC-seq). Note that this table includes 3 columns describing the Class, Major Type, and Subtype of each cell as described in a related paper analyzing snATAC data from the whole mouse brain (Li, et al. 2020. bioRxiv. https://doi.org/10.1101/2020.05.10.087585). 8. S4h - DNA methylation (snmC-seq2). Supplementary Table 5: Gene expression and epigenetic signatures per cluster Gene-by-cluster tables for each dataset based on integrated cluster analysis using the SingleCellFusion Level 2 clusters. Eight individual files for each dataset: 1. S5a - scRNA SMART 2. S5b - scRNA 10x v3 A 3. S5c - scRNA 10x v2 A 4. S5d - snRNA SMART 5. S5e - snRNA 10x v3 B 6. S5f - snRNA 10x v3 A 7. S5g - Open chromatin (ATAC-seq) 8. S5h - DNA methylation (snmC-seq2) Supplementary Table 6: Differential expression For each of the 116 consensus transcriptomic cell types, we performed differential expression (DE) analysis with respect to each of the other cell types. The table reports the top 50 conserved DE genes in each direction for ea [file 41586_2021_3500_MOESM4_ESM.zip › 2020-03-03645C-s4/Supplementary Table guide.pdf]

Supplementary Table 1: Sample parameters

List of datasets, number of cells, and other parameters of each dataset. Data from this study are available via the Neuroscience Multi-omics Archive (NEMO, RRID:SCR_016152) at https://assets.nemoarchive.org/dat-ch1nqb7.

Supplementary Table 2: Multimodal integration cluster assignments

List of all cells with cluster assignments based on 3 computational methods for integrated analysis (RNA consensus, SingleCellFusion, LIGER).

Supplementary Table 3: Cell type proportions per dataset

Proportion of each cell type within each major cell class for each transcriptomic modality.

Supplementary Table 4: Cluster analysis for individual datasets

Cluster analysis and metadata for each dataset on its own. Eight individual files:

1. S4a - scRNA SMART
2. S4b - scRNA 10x v3 A
3. S4c - scRNA 10x v2 A
4. S4d - snRNA SMART
5. S4e - snRNA 10x v3 B
6. S4f - snRNA 10x v3 A
7. S4g - Open chromatin (ATAC-seq). Note that this table includes 3 columns describing the Class, Major Type, and Subtype of each cell as described in a related paper analyzing snATAC data from the whole mouse brain (Li, et al. 2020. *bioRxiv*. https://doi.org/10.1101/2020.05.10.087585)
8. S4h - DNA methylation (snmC-seq2)

Supplementary Table 5: Gene expression and epigenetic signatures per cluster

Gene-by-cluster tables for each dataset based on integrated cluster analysis using the SingleCellFusion Level 2 clusters. Eight individual files for each dataset:

1. S5a - scRNA SMART
2. S5b - scRNA 10x v3 A
3. S5c - scRNA 10x v2 A
4. S5d - snRNA SMART
5. S5e - snRNA 10x v3 B
6. S5f - snRNA 10x v3 A
7. S5g - Open chromatin (ATAC-seq)

8. S5h - DNA methylation (snmC-seq2)

Supplementary Table 6: Differential expression

For each of the 116 consensus transcriptomic cell types, we performed differential expression (DE) analysis with respect to each of the other cell types. The table reports the top 50 conserved DE genes in each direction for each comparison. Conserved DE genes are significant in at least one dataset, while also having more than two-fold change in the same direction in all but one datasets.

Supplementary Table 7: Predicted enhancers

Enhancers predicted for each cell type based on integrated DNA methylation and ATAC-Seq data using REPTILE.

Supplementary Table 8: Integrated clusters at multiple resolutions

List of SingleCellFusion clusters at three levels of cluster resolutions (L0, L1, L2).

Supplementary Table 9: Cluster accession IDs and annotations

Cluster annotations and unique accession IDs.
